# Characterizing potential repelling volatiles for “push-pull” strategy against stem borer: a case study in *Chilo auricilius*

**DOI:** 10.1186/s12864-019-6112-4

**Published:** 2019-10-17

**Authors:** Xin Yi, Song Shi, Peidan Wang, Yaoyao Chen, Qiqi Lu, Tianyi Wang, Xiaofan Zhou, Guohua Zhong

**Affiliations:** 1 Key Laboratory of Crop Integrated Pest Management in South China, Ministry of Agriculture, South China Agricultural University, Guangzhou, China; 2 Key Laboratory of Natural Pesticide and Chemical Biology, Ministry of Education, South China Agricultural University, Guangzhou, 510642 China; 3 College of Agriculture, South China Agricultural University, Guangzhou, China; 4 Guangdong Province Key Laboratory of Microbial Signals and Disease Control, Integrative Microbiology Research Centre, College of Agriculture, South China Agricultural University, Guangzhou, China

**Keywords:** “Push-pull” strategy, Host plants, α-pinene, Olfactory

## Abstract

**Background:**

Massive techniques have been evaluated for developing different pest control methods to minimize fertilizer and pesticide inputs. As “push-pull” strategy utilizes generally non-toxic chemicals to manipulate behaviors of insects, such strategy is considered to be environmentally friendly. “Push-pull” strategy has been extraordinarily effective in controlling stem borers, and the identification of new “pushing” or “pull” components against stem borers could be significantly helpful.

**Results:**

In this study, the results of field trapping assay and behavioral assay showed the larvae of *C.auricilius*, one kind of stem borers, could be deterred by rice plant under tilling stage, its main host crop. The profiles of volatiles were compared between rice plants under two different developmental stages, and α-pinene was identified as a key differential component. The repelling activity of α-pinene against *C.auricilius* was confirmed by Y-tube olfactometer. For illuminating the olfactory recognition mechanism, transcriptome analysis was carried out, and 13 chemosensory proteins (*CSPs*) were identified in larvae and 19 *CSPs* were identified in adult of *C.auriciliu*, which was reported for the first time in this insect. Among these identified *CSPs*, 4 *CSPs* were significantly regulated by α-pinene treatment, and CSP8 showed good binding affinity with α-pinene in vitro.

**Conclusions:**

Overall, *C.auricilius* could be repelled by rice plant at tilling stage, and our results highlighted α-pinene as a key component in inducing repelling activity at this specific stage and confirmed the roles of some candidate chemosensory elements in this chemo-sensing process. The results in this study could provide valuable information for chemosensory mechanism of *C.auricilius* and for identification of “push” agent against rice stem borers.

## Background

Chemical control is considered as the most effective and efficient strategy against insect pests in agriculture [1]. However, after intensive pesticide applications, many health and environmental problems have become starkly apparent [2]. Seeking for alternative pest management strategies to reduce pesticide use and create more environmentally sound production system has aroused worldwide attentions. One such alternative method is an ecological management approach named “push-pull strategy”. By using push-pull strategy, insects are either deterred away from the main plant (push), or attracted (pull) to other areas by using stimuli that lure the insects [3]. Establishing such a system requires a comprehensive understanding of the associated chemical ecology of plant-insect, and such interactions between pests and plants are based on semiochemicals released by the plants [4]. These semiochemicals that repel pests from the main crop which is “push” component; or attract pests away from the main crop which is “pull” component [5].

Rice is one of the most important food crops, and is a staple food for more than half of the global population [6]. Rice stem borers expose great threats to rice and some graminaceous plants, as shortly after the larvae hatch, they enter into the leaf sheath to the stem by boring and making tunnel inside the stem and filling by frass to damage the plants [7]. It is difficult to achieve effective control against stem borer due to the cryptic and nocturnal habit of the adult moth and the protection provided by the host stem at its immature stages [8]. Both on-station and on-farm trials have shown that the “push-pull” strategy is effective in controlling stem borers [9]. The identification of new behaviorally active compounds released by host and non-host plants will help develop “push-pull” control strategy. The incidence of *Chilo auricilius*, one kind of stem borers, was more frequent in paddy field which located near sugarcane crop as it could rely on sugarcane for the sustenance at certain developmental stage during its life cycle [10–12]. In such case, providing that the chemicals emitted from main host plant at certain stage were potentially maladaptive and intolerable to insect, a prerequisite for insect is to adapt to alternate host plants based on its innate ability or experience [13]. During this process, identification of the vital chemicals and illuminating the mechanism of repelling process would be helpful to provide effective “push” agents to establish “push-pull” strategy against *C.auricilius* and other closely related species of stem borers [14–16].

In this study, field trapping assay was carried out to examine the number of *C.auricilius* in field at different growth stages of rice, and the indoor behavioral responses to different host plants were also recorded. To explore the mechanism of such shift in host preference, the volatile profile of rice plant at tilling stage was compared with that at seedling stage, and α-pinene was identified as a key differential compound. The repelling activity of α-pinene against *C.auricilius* was further confirmed by Y-tube assay. For the perception mechanism of *C.auricilius*, the transcriptome analysis was carried out, 19 potential chemosensory proteins were identified from adult *C.auricilius*, and the differentially expressed chemosensory genes regulated by α-pinene were identified, and eventually the binding affinity between α-pinene and the identified chemosensory protein was measured by competitive binding assay in vitro. The results in this study highlighted α-pinene as “push” component, and the mechanism of olfactory perception was also demonstrated to provide potential targets in pest management.

## Methods

### Insect

The larvae of *C.auricilius* were collected in Baiyun experimental field (114^o^44’N, 23^o^39’E) of Guangdong academy of agricultural sciences and reared with artificial diet described previously [17]. They were maintained in an incubator until emergence (condition: under temperature of 26 ± 1 °C photophase, relative humidity 70–80% with 1:1 photoperiod). Adult moths were maintained under the same conditions with a supply of 10% sucrose solution.

### Field trapping

To investigate the number of *C.auricilius* in sugarcane and paddy field at different developmental stages of rice plants, the field trapping assay was carried out in the Baiyun experimental field, Guangzhou, Guangdong of China from 4th Oct 2017 to 10th Nov 2017. First trial was conducted from 4th Oct 2017 to 10th Oct 2017. A trap device with a one-way entrance was used in the field trial. The cotton was dosed with 10% of the sucrose solution and placed inside of the trap device. The trap device was hung on the branch of rice and sugarcane crop, respectively. For each kind of crop, six trap devices were randomly placed. To eliminate the bias, the device was rotated every day to avoid uneven distribution of the insects in the field. The contents inside of the trap device were emptied every day, and each insect species was identified and the number of *C.auricilius* was counted and other species of insects were discarded. After 7 days, the total number of captured *C.auricilius* was calculated and the number of insects in each device was set as one replicate. And after 1 month, when the rice entered into the tilling stage (3rd Nov 2017 to 9th Nov 2017), the same procedure was conducted again.

### Behavioral experiments

Behavioral experiments were performed using a glass Y-tube olfactometer with a 20 cm truck length, 17 cm branch length with a 75^o^angle at the Y-junction, drying column containing activated charcoal, water column containing distilled water, sample cylinder, air pump to push air through activated charcoal and distilled water, and SA751.5 sampling pump to collect odor from plants. A humidified continuous air flow was delivered at 200 ml/min. The experiment was carried out at 25 ± 1 °C, 60 ± 5% RH, and under four 16-W cool white lights at the top to ensure even distribution of light. The rice plant and the sugarcane plant under stem elongation stage were introduced into sample cylinder after removed the mud, respectively. One insect for one time was released into the Y-tube at the entrance of the stem with maximum observation duration of 10 min per responder. The choice was recorded when the insect entered into one specific arm for at least 5 cm. Twenty insects were used in one assay, and repeated for three times.

### Headspace collection of plant volatiles

Prior to volatile collection, the root of plant was carefully covered by wet absorbent cotton. The collection method was followed by Silva. et al. described previously [18]. The plant samples (the rice plant under seedling and tilling stage) were placed in a 30 L glass jar and were left for 30 min for acclimatization prior to volatile collection. Then, a stream of charcoal filtered air was passed over the plant for 2 h at a flow rate of 20 mL min^− 1^, and volatile collection was conducted by passing the air stream for 10 h by using 200 mg Tenax TA (60/80 mesh; CAMSCO, Houston, TX, USA). The samples were analyzed by gas chromatograph (GC) coupled to a mass spectrometer (MS) from Agilent Technology Inc. The collected volatiles were released from the Tenax TA thermally at 250 °C for 5 min. During experiment, splitless mode was used with analytical column of HP-5 ms 5% Phenyl Methyl Siloxane (30 m × 0.25 mm) with array detection from 35 to 350 amu (total scan). The temperature for the transfer line was 280 °C, and the ionization energy was 70 eV. Helium was used as a carrier gas at a flow rate of 1.0 mL/min. For each treatment, three replicates were performed, and the identification process was repeated for three times to improve accuracy.

### Behavioral responses to α-pinene

The assay was carried out as previously described with little modification. Y-tube was connected to drying column containing activated charcoal, water column containing distilled water, sample bottle instead of cylinder, air pump to push air through activated charcoal and distilled water. For two sample bottles, one bottle contained 300 μL α-pinene which was diluted to 1/10 (*v/v*) with acetone prior to assay, whereas the other one contained 300 μL acetone and served as control. The recording process was carried out as previously described, and the preference index values were based on four replicates. Each replicate contained 20 larvae (3rd and 4th instar).

### Rice sample collection and expression level examinations of terpene synthases genes (TPSs)

For each sample, 50 mg rice leaves were isolated from rice plants at tilling stage and seedling stage, respectively. The samples were collected at the same time and were store at − 80 °C before RNA extraction. Total RNA was extracted from rice plants using Trizol following the instruction of Eastep®Super (Promega). The concentration of isolated RNA was measured by Nanodrop (Thermo Fisher Scientific, USA). For each sample, one μg of the isolated RNA was reversely transcribed to the first-strand cDNA by M-MLV reverse transcriptase (TaKaRa, China) and oligo(dT)_18_ as primer at 42 °C for 60 min. The expression patterns of potential *TPSs* were investigated by quantitative real-time PCR (qRT-PCR), following the protocol described previously [19].The quantitative PCR was performed using the iCycleriQ Real-Time PCR Detection System (Bio-Rad) with SYBR green dye (Taraka, China) binding to double-strand DNA at the end of each elongation cycle. The sequences of *TPSs* were isolated in “The rice annotation project database”, and the primers were designed and listed in the Additional file 1: Table S1, and tubulin was used as reference gene. All amplifications were performed with three biological and three technical replicates. Relative gene expression data were analyzed using the 2-△△CT method as described by Livak [20].

### Illumina sequencing

The larvae of *C.auricilius* at different developmental stages were mixed together and adults (5th day after emergence) were also collected. For each sample, the amount was 5 g, and repeated for three times. Total RNA was extracted by the RNA isolation kit (Omega, USA) according to the manufacturer’s instructions. The concentration of isolated RNAs was measured by Nanodrop (Thermo Fisher Scientific, USA). The procedure of cDNA library construction was conducted following the description in our previous study [21]. mRNA was purified by oligo (dT) and was then split into small fragments. The first strand of cDNA was synthesized by using a random primer and mRNA as template. Then, we used buffer for reverse transcriptase, dNTPs, RNase H and DNA polymerase I for double-strand cDNA, and the obtained cDNA was purified for end repair and poly (A) addition. Finally, the 5′ and 3′ ends of the fragments were ligated. Suitable fragments, examined by agarose gel electrophoresis, were selected as templates for PCR amplification to create a cDNA library. The cDNA library was sequenced on an Illumina sequencing platform (HiSeqTM 2000) and 100 bp paired-end reads were generated.

### Identification of chemosensory genes

*CSP* genes from larvae and adult of *C.auricilius* were identified from the de novo transcriptome assembly, respectively, and the identification process was followed as previously described [19]. TransDecoder v2.0.0 was used to annotate the coding regions in the transcriptome assembly, and the InterProScan v5 was used to check for the presence of the characteristic domain of CSPs (IPR005055) of the translated protein sequences. The TBLASTN was performed by using the identified protein sequences of CSPs as queries to identify putative CSP coding regions. Then GeneWise v2.2.0 was used to perform homology-based gene prediction by using the most similar query sequence as reference. Then all the predicted genes were further examined for the presence of the characteristic CSP domain in their translated protein sequences. A phylogenetic tree was constructed by MEGA5.0 and by maximum likelihood method. The identified CSPs in *C.auricilius* were named after orthologous genes in *Bombyx mori* [22].

### Examination of expression levels of candidate CSPs

α-pinene (≥99%) was commercially purchased from J&K® (China) under CAS number of 7785-70-8. For the insect treatment, two sets of artificial diets were provided, and one set of diet was incorporated with 1.50 mg/ml α-pinene. Another set of diet was added by acetone alone and considered as control. After 12 h starvation, two groups of 3rd instar larvae of *C.auricilius* were fed by different sets of diets, respectively. For each group, there are 15 insects, and was repeated for three times. After 24 h, all insects were collected and anesthetized. Then the insects were dissected and stored at − 80 °C before RNA extraction. The total mRNA extraction and qRT-PCR was conducted as described previously by using primers listed in Additional file 2: Table S2. All amplifications were performed with three biological replicates.

### Expression and purification for candidate CSPs

The gene-specific primers of CSP8 were designed with restriction enzyme sites and protection base attaching to the 5′ end (forward: 5′-TTATTCTGGGAYGACGAYGCCGT-3′, reverse: 5′-CGCCATGGGAAGATGAAAAGTACCCTAGC-3′). The full-length coding sequence (CDS) of *CSP8* was amplified by ExTaq DNA polymerase (TaKaRa, Japan) and was then connected to pET32a (Invitrogen, US) by T4 DNA ligase (Takara, China) at 14 °C. The product of connection was translated into pET-32a, and cultivated at 37°Cfor 14–16 h. The positive clone was identified by PCR, and after Isopropyl-D-thiogalactoside (IPTG) (1 mmol/L) was added, those positive bacterial colonies were inoculated overnight until its OD_600_ reached 0.4–0.6. The inoculated products were broken by sonic oscillator, and were examined by SDS-PAGE. The recombinant protein was purified by affinity chromatography using HisTrap columns prepacked with Ni Sepharose (GE Healthcare) as previously described [19]. The cultures were harvested by centrifugation and lysed with solution (10 mM imidazole, 300 mM NaCl and 50 mM NaH2PO4). The protein solution was washed and balanced by binding buffer in the kit (20 mM NaH2PO4, 500 mMNaCl, pH 7.8) to make sure the sample adhere to the column completely after filled the filtered solution into the column. Then, a linear gradient concentration of imidazole was used to elute the recombinant protein adhered on the column, and then purified protein was dialyzed by Tris-HCL (pH = 7.4). His-tag was removed by incubating in Bovine Enterokinase overnight. The purified protein was collected and examined by 12% SDS–PAGE. Bradford method was used to determine protein concentration [23].

### Fluorescence-based ligand binding assays

The binding affinity was measured in a 1 cm light path quartz cuvette by JASCO J-715 CD spectrophotometer (HITACHI) at room temperature. N-phenyl-1-naphthylamine (1-NPN) was used as the fluorescent probe, and all the chemicals used in the measurement were dissolved in HPLC purity grade methanol. Before measurement of binding affinity, the intrinsic florescence was examined by using protein solution in Tris-HCl buffer with different concentrations of 1-NPN (0, 2, 4, 8, 12, 16 and 20 μM). The binding affinity between CSP8 and 1-NPN was measured by recording the excitation wavelength of 2 μM 1-NPN in 50 mM Tris-HCl at 337 nm and emission spectra between 350 nm and 550 nm. After that, 2 μM of CSP8 protein was added into the solution and titrated with aliquots of 1 mM 1-NPN to final concentrations of 2 to 16 μM. The affinity between α-terpene and CSP8 were measured by competitive binding assays in presence of both protein solution and 1-NPN by adding different concentrations of α-terpene (0 to 20 μM). For each measurement, we performed three replicates. The maximum value of fluorescence emission was plotted against the concentration of α-terpene, and the curves were linearized by Scatchard plots. The dissociation constants of the competitors were calculated as described previously by using the corresponding IC_50_ values according to the equation: KD = [IC_50_]/(1 + [1-NPN]/K_1-NPN_), where [1-NPN] is the free concentration of 1-NPN and K_1-NPN_ is the dissociation constant of the protein complex/1-NPN [24].

## Results

### Field trapping

**Fig. 1:**
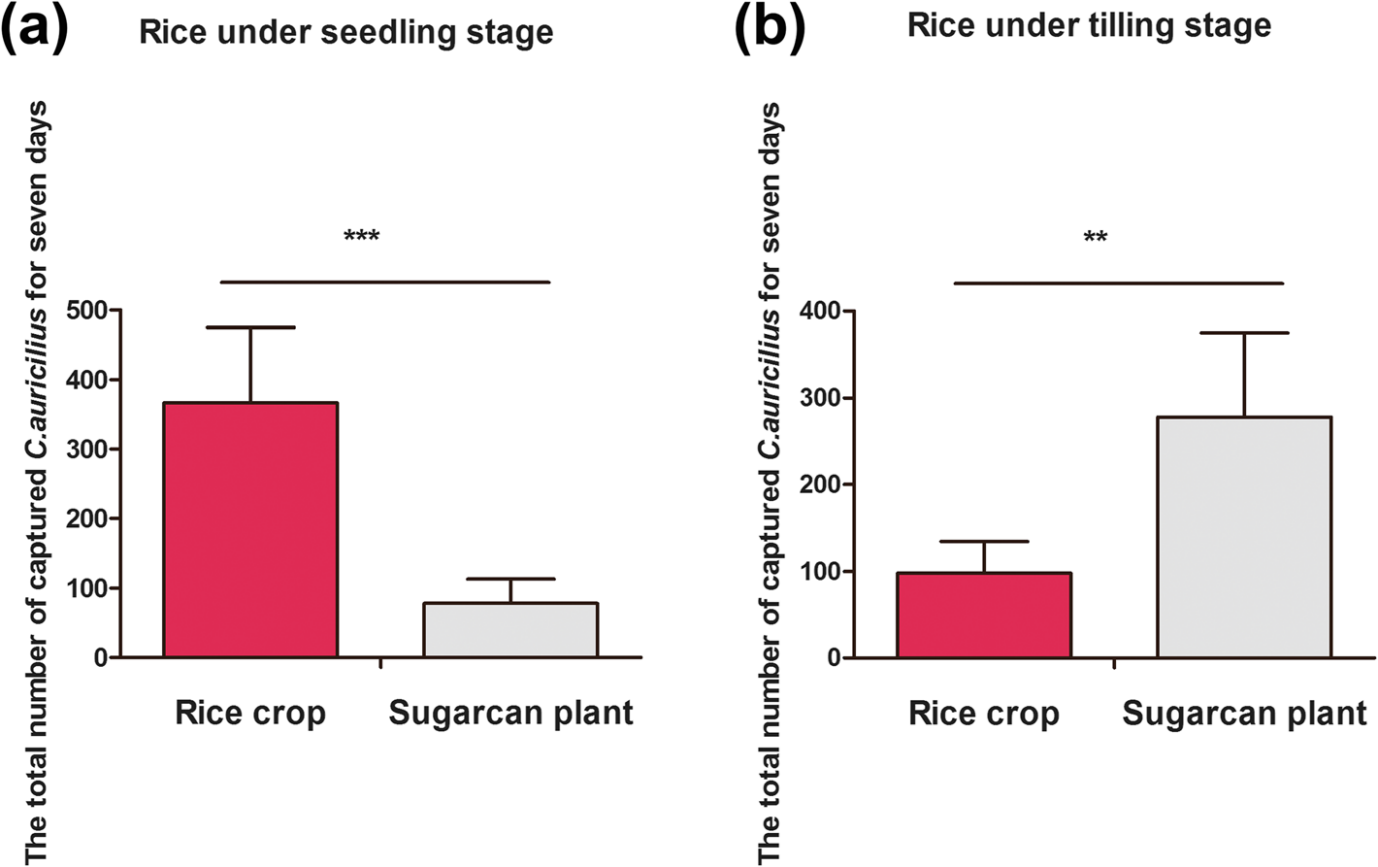
The total number of trapped *C.auricilius* for 1 week at sugarcane or paddy field. The experiment was conducted from 4th Oct to 10th Oct (**a**) and from 3rd Nov to 9th Nov (**b**), 2017 at Baiyun experimental field, Guangzhou, Guangdong, China. **a** The number of trapped insects in paddy field when the rice was under seedling stage compared with the number in sugarcane field (the sugarcane was under stem elongation stage). **b** The number of trapped insects in paddy field when the rice was under tilling stage compared with the number in sugarcane field (the sugarcane was under stem elongation stage). The number of insects in one device was served as one replicate, and there are six trapping devices in each crop field. (*t* test; ***: *P* < 0.001,**:*P* < 0.01; *, *P* < 0.05; ns, not significant)

Other than *C.auricilius*, there were many other species and they were discarded. A total of 367 ± 108 *C.auricilius* were captured for 7 days when the trap device was placed in the paddy field and the rice was under the seedling stage. And the number of captured insects at paddy field showed significant difference (*P* < 0.0001) compared with the number captured in sugarcane filed (78 ± 35) (Fig. 1a). After 30 days, when the rice plant entered into the tilling stage, the trapping assay was conducted at the same condition. However, the situation was different as 1 month ago. As the number of captured *C.auricilius* in paddy field (98 ± 36) was much less than the number in sugarcane field (278 ± 97) (Fig. 1b).

### Behavioral responses

**Fig. 2:**
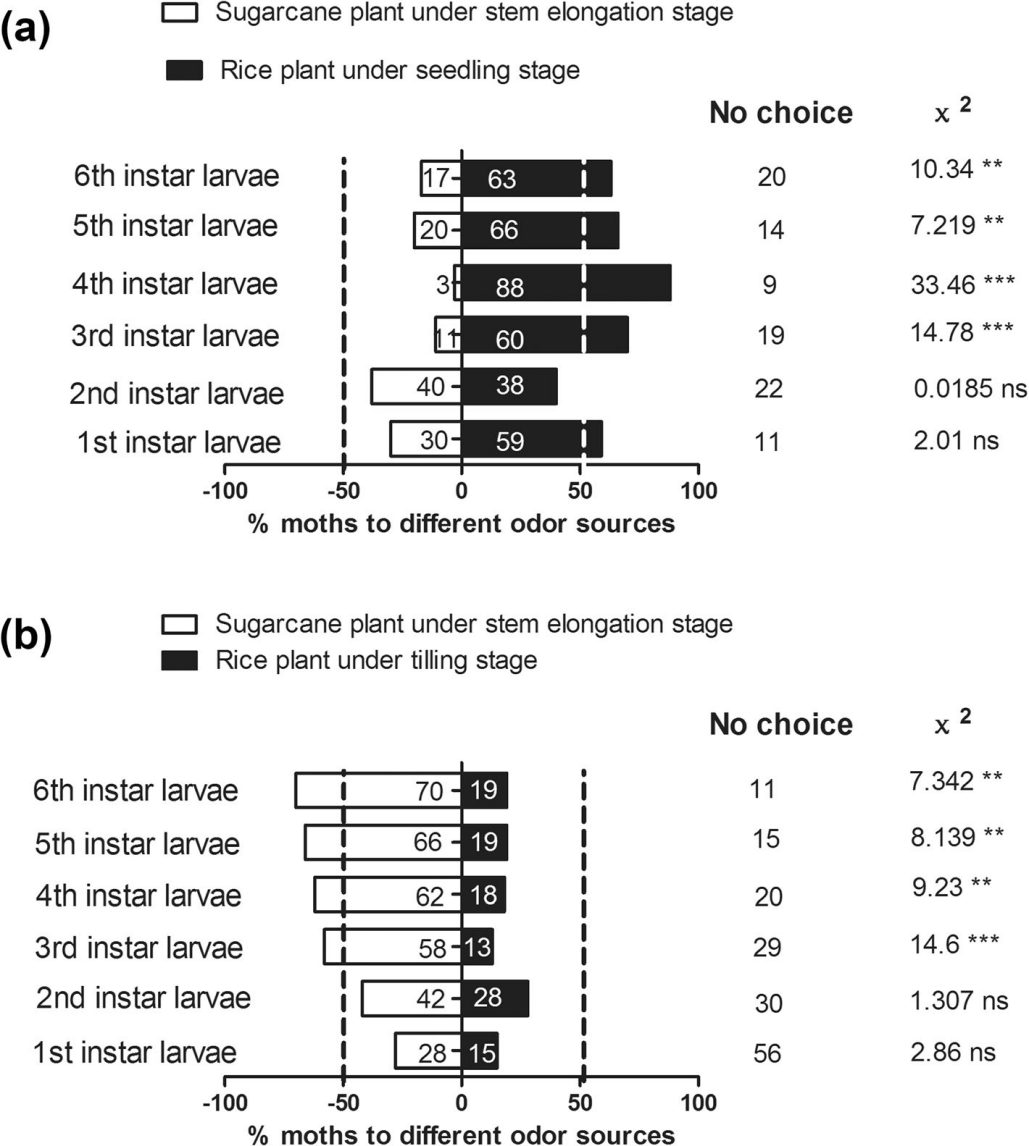
Behavioral responses of *C.auricilius* larvae to rice and sugarcane plants at different stages measured by Y-tube olfactometer. Bar represented the overall percentages of moths choosing either of the host plants (% moths to different odor sources = the number of moths choosing either arm / the total number of testing moths * 100%), numbers in bracket indicated the total number of moths choosing the arm. **a** The number of *C.auricilius* larvae choosing either sugarcane plant (under stem elongation stage) or rice plant (under seedling stage); **b** The number of *C.auricilius* larvae choosing either sugarcane plant (under stem elongation stage) or rice plant (under tilling stage). (*chi-squared* test; ***: *P* < 0.001, **:*P* < 0.01; *, *P* < 0.05; ns, not significant). No choice indicated the moth did not make choice to enter into any arm within 10 min

The preference of *C.auricilius* larvae to rice plant was compared with that to sugarcane plant by using Y-tube olfactometer. The results indicated that *C.auricilius* larvae preferred rice plant under seedling stage to sugarcane plant under stem elongation stage (3rd instar larvae, χ^2^ = 18.2, *P* < 0.0001, 4th instar larvae, χ^2^ = 33.46, *P* < 0.0001; 5th instar larvae, χ^2^ = 7.219, *P* = 0.0072, 6^th^instar larvae, χ^2^ = 10.34, *P* = 0.0013) (Fig. 2a). However, the preference of *C.auricilius* larvae changed when the rice entered into tilling stage. The *C.auricilius* larvae preferred volatiles from sugarcane to rice under tilling stage (3rd instar larvae, χ^2^ = 14.6, *P* = 0.0001; 4th instar larvae, χ^2^ = 9.32, *P* = 0.0023; 5th instar larvae, χ^2^ = 8.139, *P* = 0.0043; 6th instar larvae, χ^2^ = 7.342, *P* = 0.0067) (Fig. 2b).

### Volatile profiles of rice plants under two different stages

Eighteen compounds, including alcohols, ester, and alkane were identified from rice plant. And statistically significant differences could be observed in the concentrations of the identified volatile chemicals between the seedling and tilling stage, including linalool, tetradecane, tetradecyl vinyl ester, hexacontanoic acid, 1–4-methyl-benzene, epicedrol, pentadecane, α-pinene, cis-9-hexadecenal, (Z)- 9-octadecenamide, and 1H-indole (Table 1). Among the rice volatiles, pentadecane was the most abundant compound at seedling stage, while the amount of α-pinene was the highest compared with other volatiles at the stage of tilling. As α-pinene was reported to be as an effective repellent to insects, the following work would focus on this chemical.

**Table 1:** Major volatile compounds identified by GC-MS in rice plants under seedling and tilling stages

Compound	Retention time (min)	Relative area (%)	
		Seedling stage	Tilling stage
D-Limonene	9.9777	0.45 ± 0.26	ND
Ethylhexonol	10.1103	1.63 ± 0.22	ND ***
Undecane	12.1686	0.67 ± 0.14	0.22 ± 0.12
Tridecane	17.9126	0.92 ± 0.33	0.17 ± 0.24
Butyl dodecyl ester	19.7798	ND	0.19 ± 0.12
Tetradecane	20.5452	2.43 ± 0.14	0.32 ± 0.14 ***
Tetradecyl vinyl ester	20.7863	1.60 ± 0.64	0.37 ± 0.18 ***
Hexacontanoic acid	21.2406	0.93 ± 0.78	ND *
1–4-methyl- Benzene	22.6543	1.66 ± 0.92	0.23 ± 0.42 ***
Epicedrol	22.7757	1.44 ± 0.29	ND ***
Pentadecane	23.0311	3.13 ± 0.93	0.32 ± 0.14 ***
α-Pinene	23.6902	ND	1.37 ± 0.68 ***
cis-9-Hexadecenal	25.6299	1.63 ± 0.26	ND ***
Tridec-2-ynyl ester	25.9072	ND	0.34 ± 0.14
2-Hexyl-1-dodecanol	29.8717	0.84 ± 0.31	ND
3,4-Dihydroxyphenylglycol	36.3869	ND	0.10 ± 0.01
(Z)- 9-Octadecenamide	38.5483	2.27 ± 0.12	ND ***
1H-Indole	39.0174	0.14 ± 0.05	ND **
Total contents		20.29 ± 5.13	3.63 ± 2.19

*ND* Not detected, the data of the relative area in the table represent the mean values ± S.E.M of three examinations. Different letters indicate significant difference of the expression levels (***: *P* < 0.001, **: *P* < 0.01; *, *P* < 0.05, two-way ANOVA)

### Behavioral response to α-pinene

**Fig. 3:**
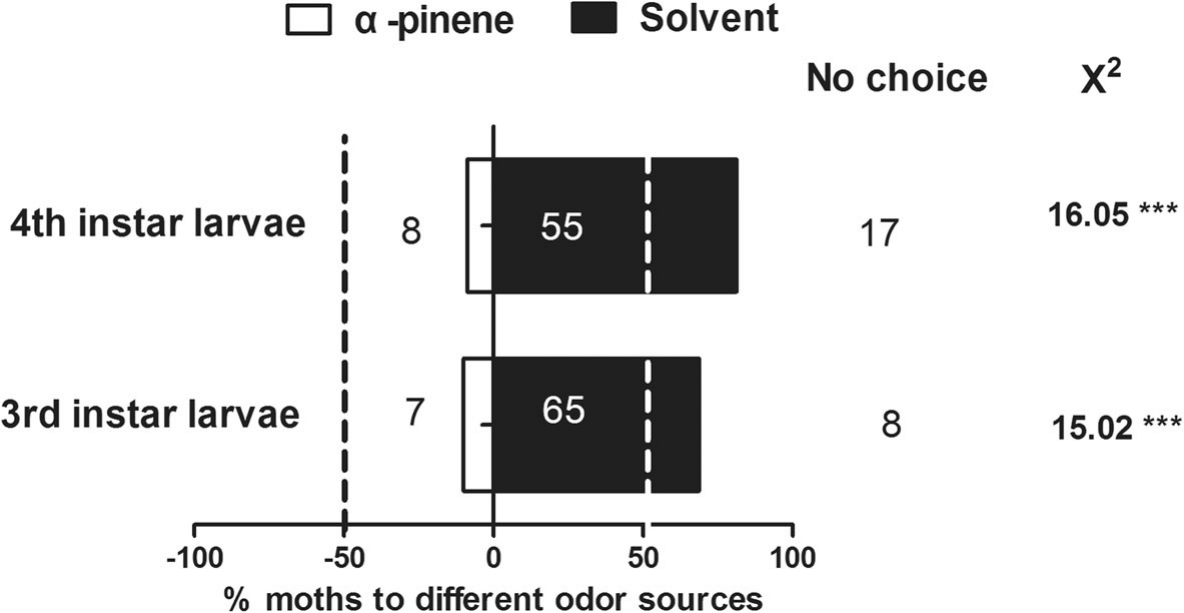
Behavioral responses of *C.auricilius* larvae to α-pinene measured by Y-tube olfactometer. 3rd and 4th instar larvae were used as candidates in the behavioral assay. Bar represented the overall percentages of moths choosing either odor arm or solvent arm (% moths to different odor sources = the number of moths choosing either arm / the total number of testing moths*100%), numbers in bracket indicated the total number of moths choosing the arm. (*chi-squared* test; ***: *P* < 0.001, **:*P* < 0.01; *, *P* < 0.05; ns, not significant). No choice indicated the moth did not make choice to enter into any arm within10 min

Then the Y-tube olfactometer was used to examine the behavioral response of *C.auricilius* to α-pinene. Based on previous observation, the 1st and 2nd instar larvae showed relatively weak mobility, therefore, 3rd and 4th instar larvae were served as testing candidates for this experiment. The results showed that the 3rd and 4th instar larvae preferred to choose the solvent arm relative to α-pinene arm, and the preference index to solvent exhibited significant difference comparing that to α-pinene (3rd instar larvae, χ^2^ = 16.06, *P* < 0.0001; 4th instar larvae, χ^2^ = 15.02, *P* = 0.0001) (Fig. 3).

### Expression pattern of TPSs

The expression patterns of *TPS* genes in rice plants under seedling and tilling stages were examined, respectively. Results showed that ten out of thirty genes exhibited significant differences between these two different growth phases. Among them, *TPS2, TPS14, TPS22*, and *TPS23* exhibited extremely distinct differences between these two stages (*P* < 0.001) (Additional file 3: Figure S1), and these genes at tilling stage expressed 19.99, 238.75, 11.25, and 5.46 fold higher than the levels at the seedling stage, respectively. All the differentially expressed *TPS* genes showed higher expression levels at the stage of tilling comparing with the expression levels at seedling stage.

### Identification of *CSPs* from *C.auricilius*

**Fig. 4:**
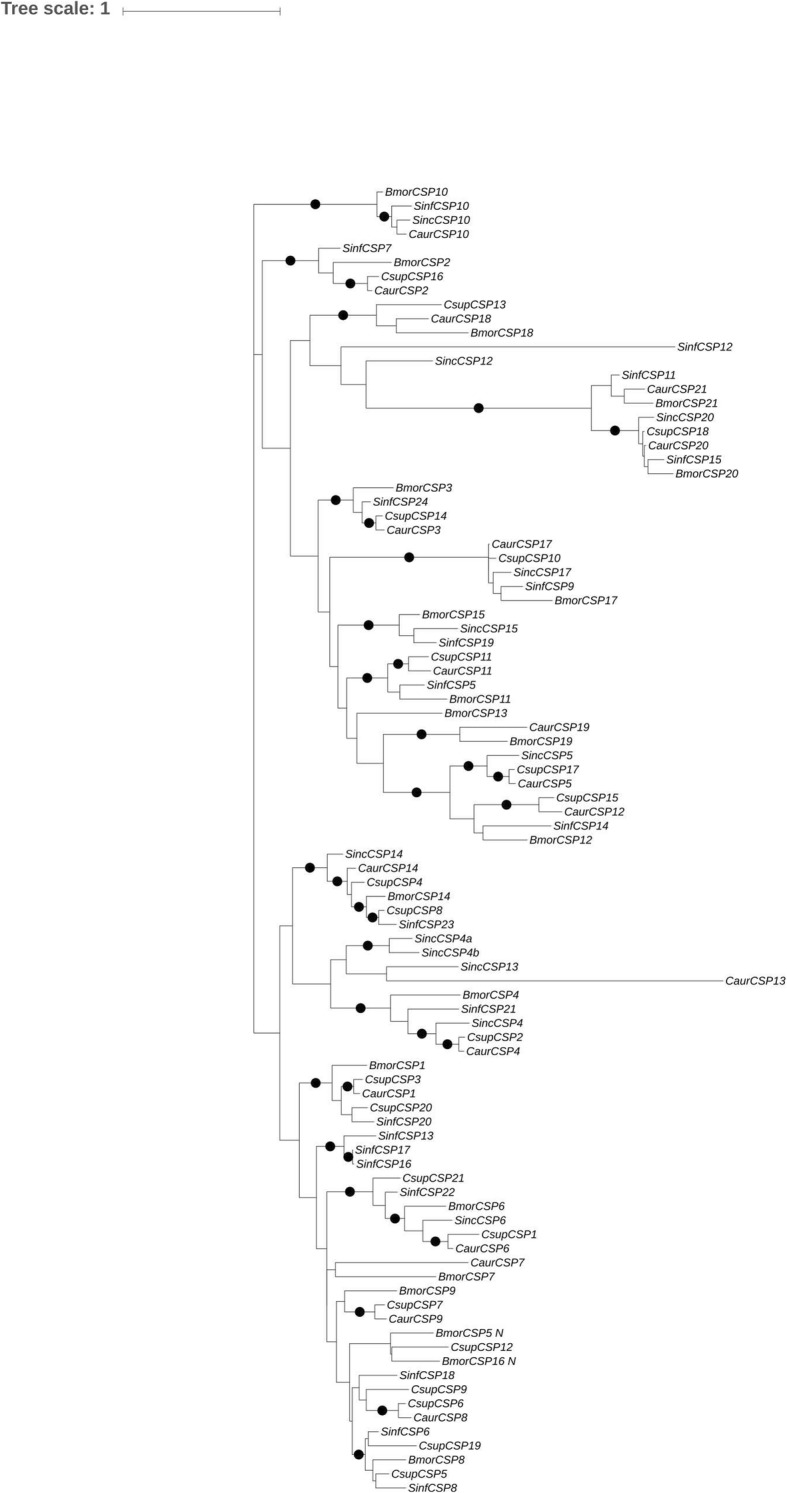
Phylogenetic analysis of *CSPs* in five Lepidopera insects. The phylogenetic tree was constructed by MEGA 5.0. Bootstrap values ≥90% (1000 replicates) were indicated as black dot at the nodes. CSPs from *C.auricilius* identified in this study were indicated as CaurCSPs. The CsupCSP, BmorCSP, SinfCSP and SincCSPs represented the CSPs identified from *C. suppressalis*, *B.mori, Scirpophaga incertulas,* and *Sesamia inferens* from previous studies [22, 25–27]

By analyzing the transcriptome assembly of *C.auricilius*, we identified 19 non-redundant *CSP* coding transcripts from adult moths (Datasheet S1, Fig. 4), which covered all the *CSPs* expressed at larvae stage. Among these identified *CSPs* in adult, six *CSPs* (*CSP2, CSP4, CSP6, CSP10, CSP13,* and *CSP18*) were not present in larvae transcriptome. The identified *CSPs* were named after the *CSPs* from *Bombyx mori* [22]. To compare the *CSPs* expressed in *C.auricilius* with that in other closely related insect species, the phylogenetic analysis was performed by using previously reported *CSPs* in *C. suppressalis* [25], *B.mori* [22]*, Scirpophaga incertulas* [26]*, Sesamia inferens* [27]*.* Phylogenetic analysis showed that all the 92 *CSPs* from the five insect species revealed well-supported clades displaying clear orthologous relationships.

### Expression variations of candidate CSPs after treated by α-pinene

**Fig. 5:**
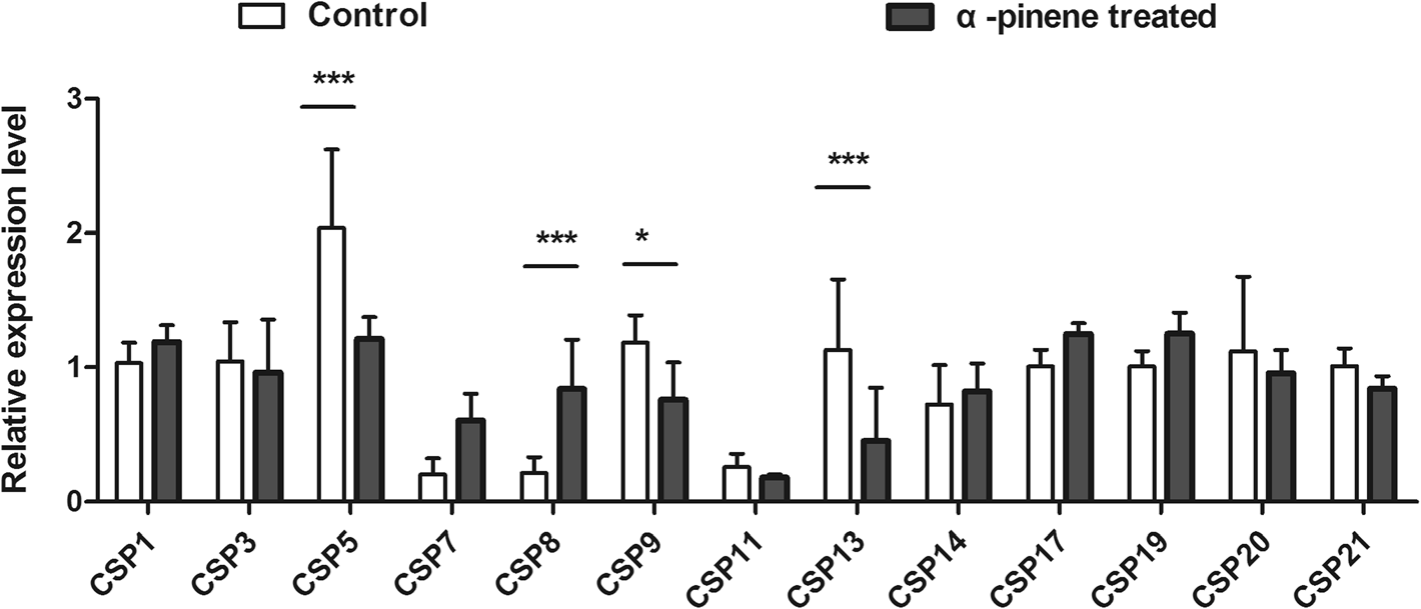
Expression levels of identified *CSPs* in 3rd instar larvae of *C.auricilius* in response to α-pinene treatments. The data represented the mean values ± S.E.M of three replicates (***: *P* < 0.001, **:*P* < 0.01; *, *P* < 0.05, two-way ANOVA)

After treated by α-pinene, the expression variations of *CSPs* in *C.auricilius* larvae in response to α-pinene were examined, the results showed that only 4 out of 13 *CSPs* exhibited significant differences in expression levels. And among these four significantly regulated *CSPs*, the expression levels of *CSP5, CSP8, CSP9,* and *CSP13* were regulated by 1.67, 3.95, 1.54, and 2.49 folds after treated by α-pinene compared with control (Fig. 5). Among these differentially expressed *CSPs, CSP8* was selected for further study as it is the only one up-regulated *CSP* after α-pinene treatment.

### Fluorescence binding assays

**Fig. 6:**
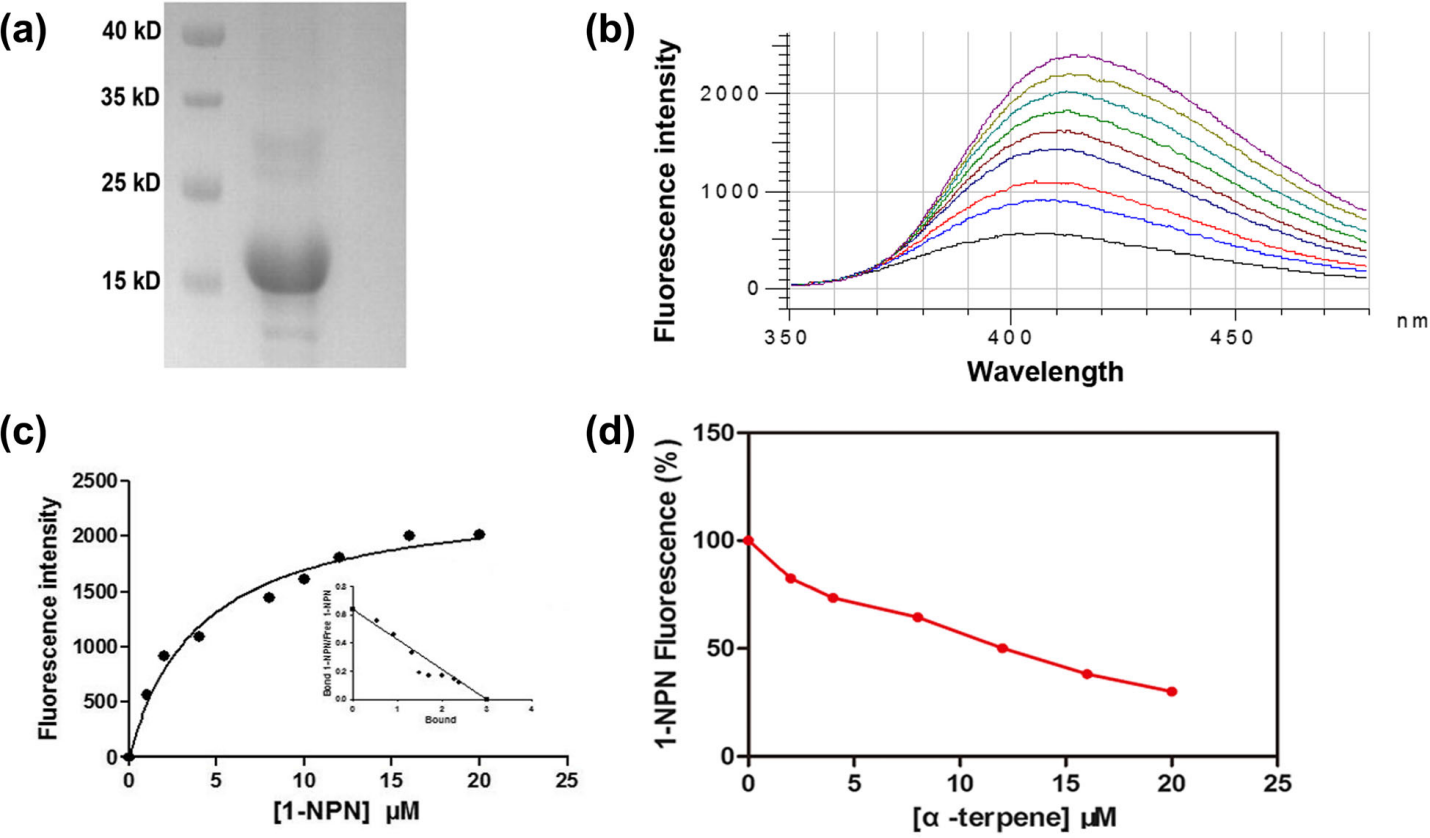
Binding affinity between CSP and α-pinene by competitive binding assay. **a** SDS-PAGE of the purified protein of CSP; M: molecular weight marker of 15, 25, 35, and 40 kDa. **b** Binding of 1-NPN to CSP. 4 μM of protein in Tris buffer was used here. And aliquots of a 1 mM methanol solution of 1-NPN were added to the protein to final concentrations of 2, 4, 6, 8, 10 12, 14, 16, 18 and 20 μM (indicated as black, blue, red, dark blue, grey, light green, dark green, yellow and purple, respectively), and the emission spectra were recorded. **c** The binding curve and the relative Scatchard plot of binding. **d** Competitive binding of α-pinene to CSP. Protein (4 μM) was incubated with 1-NPN (4 μM) and aliquots of different concentrations of ligand were added. For each set of data, fluorescence values were plotted as percent of that obtained in the absence of competitor

The CSP8 recombinant protein was induced and expressed successfully, the purified protein showed a single band with molecular weight of 18 kDa by SDS-PAGE (Fig. 6a). After the His-tag was removed successfully, the concentration for this purified solution used for further study was 5.7 mg/mL. To examine the intrinsic fluorescence, 1-NPN was added into the protein solution to make its concentration reach to 0, 2, 8, 12, 20 μM, respectively. With the increasing concentrations of 1-NPN, the intensity of fluorescence decreased from 1212 to 853, 643, 400, and 219, respectively, which indicated that 1-NPN could bind to the protein to induce fluorescence quenching. By titrating 4 μM CSP8 with increasing concentrations of 1-NPN, a saturation and linear Scatchard plot were observed (Fig. 6b&c), which indicated that CSP could bind to 1-NPN with a dissociation constant of 3.995 ± 0.13 μM, which suggested 1-NPN could be used as probe to examine the binding affinity of CSP8 to ligand. Then, 1-NPN was used as the probe to measure the binding affinity of CSP8 to α-pinene in competitive binding assay, and the results indicated that CSP8 showed high affinity for α-pinene with 11.16 of IC_50_ values (the concentration of the ligand that yielded 50% of the initial fluorescence value) and 4.34 of binding constant (Fig. 6d).

## Discussion

The indoor results of behavioral responses demonstrated that the pattern of preference of *C.auricilius* was nicely matched the situations recorded in the field. The olfactometer system, a sealed device with channels, enabled the candidate insect move freely and decide between channels permeated with the tested odor or with solve [28]. During this testing process, only the olfactory recognition was considered. Volatiles from host plants are important for insects to choose suitable sites, and differences in the types and concentrations from volatile components could directly influence the host discrimination [29, 30], the mating process [31], and reproduction of insects [32, 33]. Based on our results, we suggested that the preference shift of *C.auricilius* at specific stage could be at least partly due to the unpleasant odors emitted by rice plant at certain stage [34]. The ability to detect and discriminate toxic or unpleasant odor is essential because chemosensory conveys important information about the quality and nutritional value of food, allowing insects to avoid potentially toxic or unsuitable food and sites [14]. However, in this case, we could not rule out the possibility that the insect was attracted by the odors from sugarcane. Such scenario was not considered in this current study, and this hypothesis needs further investigation. By identifying the relevant volatiles, our results could set ground-work for further investigations on the transferring mechanism and provide valuable information for “push” odor. In this study, an expedient approach to identify semiochemicals is to compare the volatile collections from host plants at different physiological conditions [35] by using various techniques, such as solvent extraction [36], solid phase microextraction [37] or headspace collection [38], and among them, the headspace collection is preferred as it could reflect the actual releasing situation and quantities of naturally occurring compounds and it does not cause any mechanical wound on plant tissue [38]. By using different methods, the results might have significant variations in both types and concentrations of these identified volatile compounds [39]. The volatile profiles identified in this study are very similar to those obtained by Ghaninia*et al* [40], as many compounds, including ethylhexonol, tetradecane, 1,4-methyl-benzene and pentadecane, were identified in both assays. However, the quantities of volatiles differed between the present study and the previously cited one, probably because of the differences in the collection duration, different strains and different growth stages. Implied by Schlaeger *et. al.*, a complex and diverse range of compounds could be collected and identified, but only a subset of them would likely have semiochemical roles [35]. In particular, based on the results in this study, α-pinene may have the ability to repel insects, and were also consistent with results previously reported [41, 42]. As terpenes are important herbivore-induced plant volatiles involved in direct and indirect plant defense against herbivores [43]. Our study characterized the quantity of α-pinene at tilling stage was significantly higher compared with that at seedling stage of rice plant (Table 1), and behavioral assay indeed implied that *C.auricilius* preferred to choose solvent arm rather than the arm equipped with α-pinene, suggesting that α-pinene could have the ability to repel *C.auricilius* and implied its potential role to serve as a “push” component in developing “push and pull” strategy against stem borers*.* And the mRNA expression levels of many *TPS* genes showed to be significantly regulated at these two stages (Additional file 3: Figure S1). The differentially expressed *TPS* genes might contribute to the variations in proportions of α-pinene at these two different stages.

Here we show that olfaction is sufficient for an insect to differentiate unpleasant odor [13]. In the meanwhile, the preference shift of insect to food or host plants always accompanied by changes in the peripheral chemosensory system, as insect has developed a highly sophisticated sense of smell based on a variety of specific molecular elements, including odorant binding proteins and CSPs [44, 45]. By transcriptome analysis, 19 *CSPs* were identified from adult *C.auricilius* for the first time, and covered all the *CSPs* expressed in larvae (13 *CSPs* in larvae). There is possibility that some of the *CSPs* could not be identified in this study due to the limit of quality of transcriptome sequencing, assemble or annotation, and expression abundance in the insect body [46]. Analyzing expressions of chemosensory receptors after odorant exposure can help identify ligand–receptor interactions in vivo*,* as it was discovered that exposure to artificially high concentrations of odorants could lead to reliable alterations in mRNA levels of interacting odorant receptors in mammal and insects [47]. After treated by 1.50 mg/ml α-pinene, 4 *CSPs* showed significant variations in larvae *C.auricilius*, suggesting their roles in detection this chemical. High degree binding affinity between CSP8 and α-pinene in vitro was observed by competitive fluorescent binding assay (Fig. 6). The binding between protein and ligand was attributed to the spatial structure of proteins and ligands, especially their specific interactions. The binding affinity established in this study does not necessarily mean the actual binding and activation in the lymph of insect, therefore, other techniques, such as RNA interference and knockout, would be essential to fully confirm such binding and its repelling activity also should be evaluated in the field prior to actual applications. And at the same time, those down-regulated and unchanged CSPs might also have roles and contributed to perception of α-pinene, which needs further investigation.

## Conclusions

In this study, the preference shift of *C.auricilius* to rice plant at certain stage was confirmed, α-pinene was identified as a key repellent compound and its repelling activity against *C.auricilius* was also evaluated by Y-tube olfactometer. The transcript levels of 4 *CSPs* were found to be differentially regulated after the treatment of α-pinene, and good binding affinity was confirmed in vitro by competitive binding assay, which suggested such protein was potentially responsible for the perception of α-pinene [48]. Overall, our results could help shed lights on the recognition mechanism of *C.auricilius* to rice plants and α-pinene could serve as an important “push” agent in developing push-pull strategy against *C.auricilius* and other closely related stem borers [49].

## Supplementary information

**Additional file 1:** **Table S1.** The primers used in this study to examine the expression level of terpene synthase genes.

**Additional file 2:** **Table S2.** The primers used in this study to examine the expression level of CSPs responding to α-pinene.

**Additional file 3:** **Figure S1.** Variations in expression levels of *TPS* genes at seedling stage and tilling stage of rice plants. The data represented the mean values ± S.E.M of three replicates (***: *P* < 0.001, **:*P* < 0.01; *, *P* < 0.05, one-way ANOVA).

## Data Availability

Original fastq files are available at NCBI Sequence Read Archive with BioProject reference PRJNA563117.

## Abbreviations

1-NPN: N-phenyl-1-naphthylamine
CDS: Coding sequence
CSP: Chemosensory protein
GC: Gas chromatograph
IPTG: Isopropyl-D-thiogalactoside
MS: Mass spectrometer
qRT-PCR: Quantitative real-time PCR
TPSs: Terpene synthases genes
