# Supplementary material for: Characterizing potential repelling volatiles for “push-pull” strategy against stem borer: a case study in Chilo auricilius
Source: BMC Genomics. 2019 Oct 17;20:751. doi: 10.1186/s12864-019-6112-4 (PMC6796385; doi:10.1186/s12864-019-6112-4)
Supplement: Supplementary file 1 — Additional file 1: Table S1. The primers used in this study to examine the expression level of terpene synthase genes. [file 12864_2019_6112_MOESM1_ESM.docx]

Table S1 The primers used in this study to examine the expression level of terpene synthase genes

| Primer name | Sequence(5’-3’) | Primer name | Sequence(5’-3’) |
| --- | --- | --- | --- |
| OsTPS1-qForward | CAGTTGTTCCTGCTCGACCTC | OsTPS2-qForward | CGGAGGCGGAGATAAGTGA |
| OsTPS1-qReverse | CAGTTGGCGGCTCATAGTTG | OsTPS2-qReverse | AGGGGAATAGGGTTGACGAA |
| OsTPS3-qForward | GAAATACCAGTTGCCAGGGAC | OsTPS4-qForward | ATCTTCGTCCGTGCGTTTG |
| OsTPS3-qReverse | GATCTCATGCAGCTAGGTAAGGA | OsTPS4-qReverse | TTCATTGGGCCATTTCGTG |
| OsTPS5-qForward | CATTCTGCCCAATCCCTTTAC | OsTPS6-qForward | CAACAGACTCGACCAGCTACATT |
| OsTPS5-qReverse | CGAACCTCTTTCACCCATCTT | OsTPS6-qReverse | CGATAACAGCCGTCAACCAA |
| OsTPS7-qForward | GGGCACATCTCCAAGGTTTT | OsTPS8-qForward | TATCGCCGAATCGTGGTG |
| OsTPS7-qReverse | TTTTCGCAGTCATCACTTTACG | OsTPS8-qReverse | TCGCAAGGTTCATTAGGTGG |
| OsTPS9-qForward | CCGTGGACTCCTTTGCTGT | OsTPS10-qForward | CACGAGCAAACATACAACCCC |
| OsTPS9-qReverse | CCTCGCTCCTGAAAACCTC | OsTPS10-qReverse | TTGAAAGATCCTCCCACCACT |
| OsTPS11-qForward | AGCAAGAGGACGAACACGAC | OsTPS12-qForward | GGAAGCTCTAAAGGGGCAAGT |
| OsTPS11-qReverse | AGGGACAGCAATCCAAATACC | OsTPS12-qReverse | CGGAATCGGAGGGAAGTAAC |
| OsTPS13-qForward | CTTGGAGCCAGATGAGAAGC | OsTPS14-qForward | TGACGTGATGCAACGGTTTT |
| OsTPS13-qReverse | CCACAGCCATTGTTGGGTAG | OsTPS14-qReverse | GCACCCTTTTCATCCCACC |
| OsTPS15-qForward | TTCTGCTATGTCGCCTACTGC | OsTPS16-qForward | ACAGTAATGGGTCGTGGGG |
| OsTPS15-qReverse | TCTATAAAGGTCGGGAATTGGT | OsTPS16qReverse | GCAGGGAATGTGAGGTTGAA |
| OsTPS17-qForward | TGGAGGACGAATGGAAAACA | OsTPS18-qForward | CTCGGGTCGCAGAACAAA |
| OsTPS17-qReverse | CCGTAGAACAACTGGATGCC | OsTPS18-qReverse | AGCACATCACGGTGGTCAAT |
| OsTPS19-qForward | TCCTTGTCCATGATGAGCCA | OsTPS20-qForward | TTTGGTTGGCTAAACACTTCC |
| OsTPS19-qReverse | TTGAGCCTACTTCCGTCCCT | OsTPS20-qReverse | CTCTGCTTGGTCATTGCATAGT |
| OsTPS21-qForward | CGCTACCGCATTCCTACCA | OsTPS22-qForward | TGGAGGTCAGACGAATGGAT |
| OsTPS21-qReverse | CGCTTGTGACCTTGTTCTCATT | OsTPS22-qReverse | AGCAAGTGAAACCGAAGAGC |
| OsTPS23-qForward | TTCGGTTTCGCCTGCTTA | OsTPS24-qForward | GGAGCACGGCTTATGGGTA |
| OsTPS23-qReverse | CTTGGGTCATTCGCTATCTCA | OsTPS24-qReverse | TGTTAAGGGCACGCTTGACT |
| OsTPS25-qForward | GATCAGCAACTACGTGCCTACA | OsTPS26-qForward | TAGGAAACCTGTTGGAGTGGG |
| OsTPS25-qReverse | TGGTCCGACAAGATACAAAGC | OsTPS26-qReverse | TGTGGCAGCAGTTGTGGAA |
| OsTPS27-qForward | CTTCTTCGACGTTGGAGGATC | OsTPS28-qForward | CAACGCAGCTCACCTTCTAAC |
| OsTPS27-qReverse | CTGCTGTTGGCACATACCG | OsTPS28-qReverse | GGTCCTTGGTAACGGTATCTCA |
| OsTPS29-qForward | TGAAGGTGCAGATCAGGTAGC | OsTPS30-qForward | AGGAAGGTAGTAAAGGGCTCG |
| OsTPS29-qReverse | TCATCGGTTGTGACATCGTTT | OsTPS30-qReverse | CTTTATCGTTGTAGTCGGTGTTG |
| OsTPS31-qForward | CAATGAAGACGATGAGCGAAAG | OsTPS32-qForward | TACTGACGGCTGTGGTTGATG |
| OsTPS31-qReverse | GCAGTAGGTGGCTGGTAACTGA | OsTPS32-qReverse | CGATACGCTTGGTCGTGTTG |
| OsTPS33-qForward | AGGTGTCTGGAGCGTTTGC | Tubulin-qForward | TGAGTTCTCCGATACGATTGAC |
| OsTPS33-qReverse | GGACTCTGGCGAGGTATTGC | Tubulin-qReverse | TGTTATAATCCGCGAGAGTACG |
