# Supplementary material for: Characterizing potential repelling volatiles for “push-pull” strategy against stem borer: a case study in Chilo auricilius
Source: BMC Genomics. 2019 Oct 17;20:751. doi: 10.1186/s12864-019-6112-4 (PMC6796385; doi:10.1186/s12864-019-6112-4)
Supplement: Supplementary file 2 — Additional file 2: Table S2. The primers used in this study to examine the expression level of CSPs responding to α-pinene. [file 12864_2019_6112_MOESM2_ESM.docx]

Table S2 The primers used in this study to examine the expression level of CSPs responding to α-pinene

| **Name** | **Sequence** | **Name** | **Sequence** |
| --- | --- | --- | --- |
| CSP1-qForward | GCAGTTGGTGATGGCTTTGAGT | CSP3-qForward | TCATGGTTGATGAGATGCCCTA |
| CSP1-qReverse | AAGATGAAGTGCATTGTGGTCCTA | CSP3-qReverse | GGTGCCTTATGTAAACTGTGCG |
| CSP5-qForward | TTCTCCTGGAATCCCTTGACC | CSP7-qForward | ATCTCCGGTAGGGCTTGCTT |
| CSP5-qReverse | TCGTATCGTTGTTTGCTGTGC | CSP7-qReverse | TTGTGCCTGTTTCTTGACTTTGAC |
| CSP8-qForward | GCCAGTAGTCCTGCTCGTTGTT | CSP9-qForward | CCACTTACATCTCAACTCCTTTCC |
| CSP8-qReverse | GCCAAGTGTACTCCGACCCA | CSP9-qReverse | TCGTCCAGAGCAGTACACCG |
| CSP11-qForward | GGGTAGTTTCGTTGAATGTAGGC | CSP13-qForward | GCGTAGTCCTGTGGCAGTTTC |
| CSP11-qReverse | CAGATGACAGACGCTCAGTTGG | CSP13-qReverse | CTGAGAACGATGGTTACGATGTG |
| CSP14-qForward | CCTGTGGCAGTTTCTCCTTGAT | CSP17-qForward | CCAGTAGTCCTGCTCGTTGTTG |
| CSP14-qReverse | AGCTGTTGCTTTGGCATACGT | CSP17-qReverse | GCCAAGTGTACTCCGACCCA |
| CSP19-qForward | CAGACACTTGCGGTCGTTGA | CSP20-qForward | CCTTCCAATAGTCTGGATGTTTGT |
| CSP19-qReverse | ATACCAGCACAAAAGGGTTCAA | CSP20-1Reverse | CTCCCTGATGCCTTAGAGCAC |
| CSP21-qForward | CTTCTCCAACGCACAGTTTACAT | Actin-Forward | GCTACCCTGTCAGTCCACCAG |
| CSP21-qReverse | CGGTACGACAGCATTAACATCC | Actin-Reverse | GAAACCGGGCATGAAGAAGTG |
