# Supplementary figures and images for: Characterizing potential repelling volatiles for “push-pull” strategy against stem borer: a case study in Chilo auricilius
Source: BMC Genomics. 2019 Oct 17;20:751. doi: 10.1186/s12864-019-6112-4 (PMC6796385; doi:10.1186/s12864-019-6112-4)

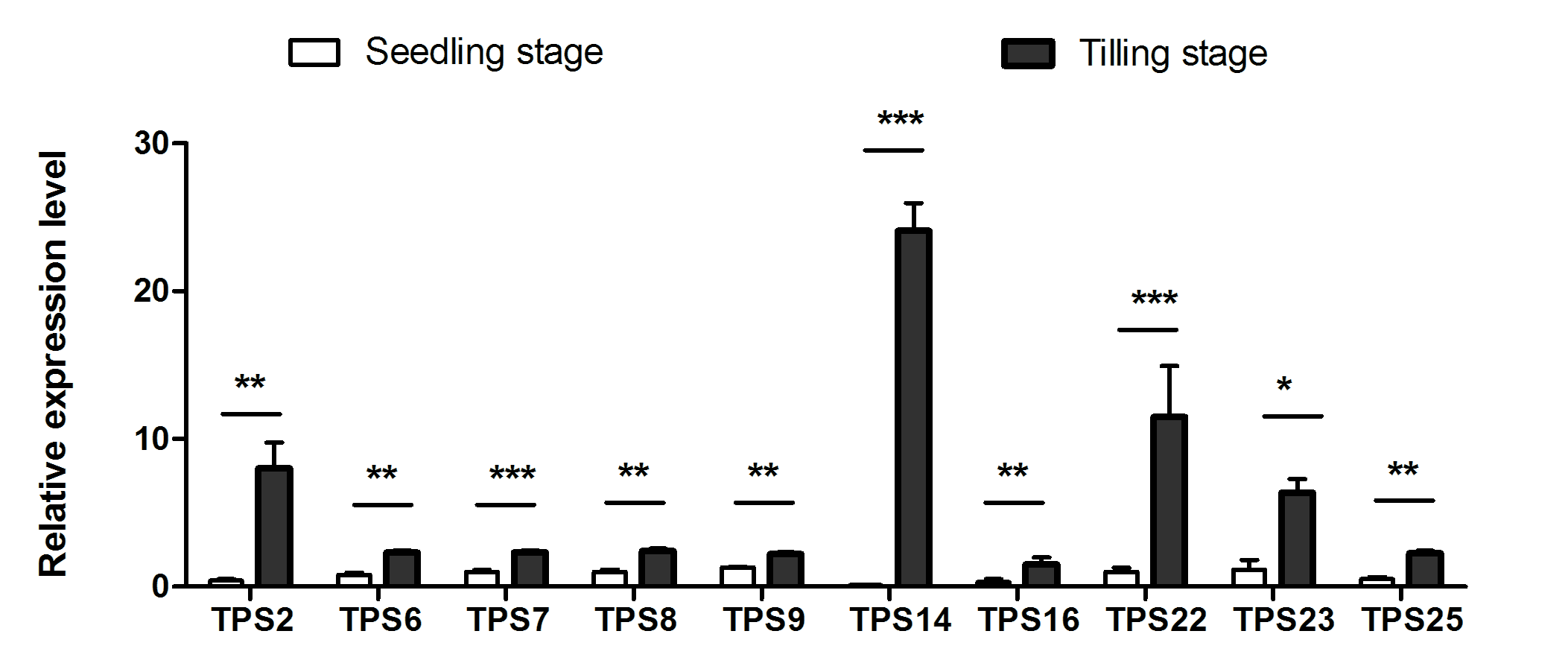

Supplement: Supplementary file 3 — Additional file 3: Figure S1. Variations in expression levels of TPS genes at seedling stage and tilling stage of rice plants. The data represented the mean values ± S.E.M of three replicates (***: P < 0.001, **:P < 0.01; *, P < 0.05, one-way ANOVA). [file 12864_2019_6112_MOESM3_ESM.tif]
